# Concentration polarization phenomenon in the case of mechanical pressure difference on the membrane

**DOI:** 10.1007/s10867-017-9448-5

**Published:** 2017-05-12

**Authors:** Sławomir Grzegorczyn, Andrzej Ślęzak, Brygida Przywara-Chowaniec

**Affiliations:** 1 Department of Biophysics, School of Medicine with the Division of Dentistry in Zabrze, Medical University of Silesia, 19 H. Jordan Str., 41808 Zabrze, Poland; 2 Institute of Health and Nutrition Sciences, Department of Biophysics, Częstochowa University of Technology, 36B Armia Krajowa Al, 42200 Częstochowa, Poland; 3 2nd Department of Cardiology, School of Medicine with the Division of Dentistry in Zabrze, Medical University of Silesia, 10 Curie-Skłodowskiej Str., 41–800 Zabrze, Poland

**Keywords:** Membrane transport, Concentration polarization, Bacterial cellulose membrane, Mechanical pressure difference, Kedem–Katchalsky equations

## Abstract

We analyzed the transport of KCl solutions through the bacterial cellulose membrane and concentration boundary layers (CBLs) near membrane with pressure differences on the membrane. The membrane was located in horizontal-plane between two chambers with different KCL solutions. The membrane was located in horizontal-plane between two chambers with different KCL solutions. As results from the elaborated model, gradient of KCL concentration in CBLs is maximal at membrane surfaces in the case when pressure difference on the membrane equals zero. The amplitude of this maximum decreases with time of CBLs buildup. Application of mechanical pressure gradient in the direction of gradient of osmotic pressure on the membrane causes a shift of this maximum into the chamber with lower concentration. In turn, application of mechanical pressure gradient directed opposite to the gradient of osmotic pressure causes the appearance of maximum of concentration gradient in chamber with higher concentration. Besides, the increase of time of CBLs buildup entails a decrease of peak height and shift of this peak further from the membrane. Similar behavior is observed for distribution of energy dissipation in CBLs but for pressure difference on the membrane equal to zero the maximum of energy dissipation is observed in the chamber with lower concentration. We also measured time characteristics of voltage in the membrane system with greater KCl concentrations over the membrane. We can state that mechanical pressure difference on the membrane can suppress or strengthen hydrodynamic instabilities visible as pulsations of measured voltage. Additionally, time of appearance of voltage pulsations, its amplitude, and frequency depend on mechanical pressure differences on the membrane and initial quotient of KCl concentrations in chambers.

## Introduction

Ag|AgCl electrodes dipped directly into electrolyte solutions in a membrane system allow measuring the potential difference between these points. Electrical potential differences depend on the concentrations of ions in these points and transport properties of membrane. By appropriate selection of points, we can characterize phenomena appearing in the membrane system such as diffusion and hydrodynamic instabilities [1–3]. We used bacterial cellulose membranes for modeling of phenomenon of concentration polarization of membrane because this membrane does not have ions bounded with its structure. Water, small molecules, and ions permeate through this membrane. Besides, this membrane is not permeable to bacteria. These properties of the bacterial cellulose membrane render it an effective cover for hard to heal wounds. The microenvironment at wound surface under the cover made from bacterial cellulose membrane accelerates healing process of wound [4–6]. The concentration polarization of the membrane reduces transport of solute through the membrane [7–9]. Different elaborated techniques, such as stirring of solutions or flow of permeate and feed solutions along the membrane decrease influence of this phenomenon on membrane transport. The main structures observed during concentration polarization of the membrane are concentration boundary layers (CBLs), which are thin layers adjacent to membrane surfaces. CBLs are characterized by their thickness and gradient of concentration in CBLs [10, 11]. Buildup of CBLs depends on the membrane transport properties and diffusion coefficient of solute in solutions [1, 12, 13]. Additional thermodynamic forces, such as mechanical pressure difference and difference of electrical potential on the membrane, modify the structure of CBLs, and thereby change the transport conditions for solutes [14]. Applications of these thermodynamic forces in desalination and purification of water change the degree of concentration polarization of membrane and kinetics of CBLs buildup. The increase of efficiency of processes of reverse osmosis and power generation in membrane systems is an example of problems of suitable application of thermodynamic forces on the membranes [15, 16].

In a previous article, we elaborated upon the model of difference equations for transport of solute through the membrane and CBLs. We showed [17] that measured and modeled voltage between electrodes in the membrane system with lower solution density over the membrane (configuration A of the membrane system) is asymmetric because of pressure differences on the membrane. Based on this model, in this article we present the results of calculations for distribution of KCl concentration in configuration A of the membrane system with difference of mechanical pressure through the membrane. The presented graphs show that applied difference of mechanical pressure through the membrane changes the symmetry of distribution of KCl concentration in chambers of the membrane system. The consequence of this is different thicknesses of CBLs in both chambers. A blurry CBL (with greater thickness and lower gradients of solute) appears in the chamber with lower pressure while compressed CBL in the chamber with higher pressure. In turn, for configuration of the membrane system with higher-density solution over the membrane (configuration B), we calculated and measured the time of CBLs buildup needed for appearance of convective stirring of solutions near the membrane caused by gravitation. This time also depends on the difference of mechanical pressure through the membrane.

## Theory

To describe the transport of electrolyte through a membrane, we used the Kedem–Katchalsky equations written in the general form [18]

1 $$ {J}_v={L}_p\left(\varDelta P-{\displaystyle \sum_{j=1}^n{\sigma}_j\varDelta {\pi}_j}+\beta i\right) $$

2 $$ {J}_s={\overline{C}}_s\left(1-{\sigma}_s\right){J}_v+{\displaystyle \sum_{j=1}^n{\omega}_{s j}\varDelta {\pi}_j}+\frac{t_s}{z_s F} i $$

3 $$ i=-\kappa \cdot \beta \cdot {J}_v+\kappa \cdot {\displaystyle \sum_{j=1}^n\frac{t_j}{z_j F}\frac{RT}{C_j}\varDelta {C}_j}+\kappa \cdot E $$

where *J*_*v*_ and *J*_*s*_ are the volume and ion fluxes (*s* – indexes for suitable ions, *n* number of ions in solution), *i* is the density current through the membrane, *ΔP* = *P*_*h*_ − *P*_*l*_ is the difference of mechanical pressure through the membrane, *Δπ*_*s*_ = *RT*(*C*_*h*_ − *C*_*l*_) is the osmotic pressure difference, ${\overline{C}}_s=\left({C}_h-{C}_l\right){\left[ \ln \left({C}_h{C_l}^{-1}\right)\right]}^{-1}$ is an average ions concentration in the membrane and $E=\frac{\varDelta \tilde{\mu}}{z_s F}$ is the gradient of electrical potential on the membrane. *C*_*h*_ and *C*_*l*_ (*C*_*h*_>*C*_*l*_) are the ion concentrations in chambers, *L*_*p*_,*σ*_*s*_ and *ω*_*s*_ are hydraulic permeability, reflection and solute permeability coefficients for membrane suitably. *β*, *t*_*s*_ and *κ* are electroosmotic coefficient, transference number of ions *s* and conductivity of the membrane. Besides *F*, *R*, and *T* are Faraday number, gas constant, and absolute temperature respectively.

In turns, transport of solute through the CBLs can be described by equation

4 $$ \frac{\partial C}{\partial t}=-\frac{\partial {J}_s}{\partial x}={D}_s\frac{\partial^2 C}{\partial {x}^2}-\mathrm{v}\frac{\partial C}{\partial x} $$

where *D*_*s*_ is the diffusion coefficient of solute and v is the velocity of solution movement in chambers. For measurement of voltage in the membrane system by means of millivoltmeter with high input resistance, we assumed that density current through the membrane equals zero. Using the above assumption and Eqs. (1)–(4), we get the difference equations for transport of solute in CBLs in the membrane system [17] written in new, shortened form

5 $$ {C}_{i, n}^{k+1}={C}_{i, n}^k+\frac{\varDelta t}{d_w}{\chi}_{i, n}^k-\frac{\varDelta t}{d_w^2}{D}_s{\gamma}_{i, n}^k $$

6 $$ {\chi}_{i, n}^k=\left|\begin{array}{c}\hfill \begin{array}{l}\left({A}^k+{B}^k RT\left({C}_{1,1}^k-{C}_{2,1}^k\right)\right){\left(-1\right)}^i-\left({C}_{i,1}^k-{C}_{i,2}^k\right){L}_p\varDelta P\kern1em  for\kern0.5em  n=1\\ {}\end{array}\hfill \\ {}\hfill {\left(-1\right)}^i\left({C}_{i, n}^k-{C}_{i, n-1}^k\right){L}_p\varDelta P\kern1em  for\kern0.5em  n>1\hfill \end{array}\right. $$

7 $$ {\gamma}_{i, n}^k=\left|\begin{array}{c}\hfill \left({C}_{i,1}^k-{C}_{i,2}^k\right)\kern1em  for\kern0.5em  n=1\hfill \\ {}\hfill \hfill \\ {}\hfill \left({C}_{i, n+1}^k+{C}_{i, n-1}^k-2{C}_{i, n}^k\right)\kern1em  for\kern0.5em  n>1\hfill \end{array}\right. $$

where *C*_*i*,*n*_^*k*^ are the concentrations in suitable layers (second subscript is for layers *n* = *1*,2,..), in the chamber with lower concentration (first subscript *i* = 2) and in the chamber with higher concentration (first subscript *i* = 1) at time *k*. ${\overline{C}}^k=\left({C}_{1,1}^k-{C}_{2,1}^k\right)\cdot {\left[ \ln \left({C}_{1,1}^k{\left({C}_{2,1}^k\right)}^{-1}\right)\right]}^{-1}$ is the average concentration in the membrane, ${A}^k=\left(1-{\sigma}_s\right){\overline{C}}^k{L}_p\varDelta P$ and ${B}^k={\omega}_s-{\sigma}_s{L}_p\left(1-{\sigma}_s\right){\overline{C}}^k$. Moreover, *d*_*w*_ is the thickness of the layer and Δ*t* is a time interval used in the recursive method of solution of differential equations. We also assumed that the velocity of solution movement in chambers is equal to the volume flux through the membrane v = *J*_*v*_ = *L*_*p*_ *ΔP*.

Using difference equations (5)–(7), we calculated distribution of concentrations in chambers of the membrane system with applied gradient of mechanical pressure on the membrane. Besides analyzing the changes in time of concentration distribution in chambers, we also calculated CBL thicknesses based on the criterion [19].

8 $$ \frac{C_o-{C}_{\delta}}{C_o-{C}_m}= K $$

where *C*_o_, *C*_m_, and *C*_δ_ are the solute concentrations in chamber at initial moment, at membrane surface and at the distance δ from the membrane respectively. δ is the thickness of CBL. In our calculations, we assumed *K* equal to 0.01 [19, 20].

In turn, the dissipation energy is the main thermodynamic parameter characterizing loss of energy during transport of solute in the membrane system. The dissipation energy is defined as $\varPhi ={\displaystyle \sum_s{J}_s{X}_s}$ [18] and for transport of solute through CBLs can be written as

9 $$ \varPhi ={J}_s\cdot \varDelta {\mu}_s={D}_s\frac{\partial C}{\partial x}\cdot \frac{1}{C} RT\varDelta C $$

where *μ*_*s*_ is the chemical potential of solute s. Writing dissipation energy for CBLs described by Eq. (9) in the form of difference equations for layers (*n*) in the chambers with higher (*i* = 1) and lower (*i* = 2) concentration at time *k* we get

10 $$ {\varPhi}_{i, n}^k=\frac{D_s}{d_w}\cdot \frac{1}{C_{i, n}^k} R T{\left({C}_{i, n}^k-{C}_{i, n-1}^k\right)}^2 $$

The distribution of energy dissipation in CBLs can be calculated using Eqs. (5)–(7) and (10). For configuration of the membrane system with higher density of solution over the membrane (configuration B), transport of solute through the membrane causes the appearance of density gradients near membrane, oppositely directed to gravitational acceleration. Sufficiently high values of density gradients in CBLs cause the appearance of hydrodynamic instability in the membrane system [21]. Hydrodynamic instability is the movement of solution with higher density downward and with lower density upward (gravitational convection). This phenomenon causes a blur of CBLs and a decrease of concentration gradients in CBLs. The critical value of concentration Rayleigh number allows identification of moment of appearance of hydrodynamic instability in CBLs [22]. The concentration Rayleigh number for CBL can be written as [23, 24]

11 $$ R a=\frac{g\frac{\partial \rho}{\partial C}\varDelta C{\delta}^3}{\rho \nu {D}_s} $$

where *g* is the gravitational acceleration, ΔC is the difference of concentrations in CBL, *δ* is the CBL thickness, *ρ* is the density of solution, *ν* is the kinematic viscosity coefficient for solution, and *D*_s_ is the diffusion coefficient for solute in solution. Analyzing CBL stability we should take into consideration that conditions of gravitational convection in the membrane system are of the type: rigid surface (membrane surface) and free surface (border of CBL in chamber). In this case, the critical value of Rayleigh number for CBL is equal to (*Ra*)_c_ = 1100.6 [25]. For the membrane system in B configuration and for *Ra* < (*Ra*)_c_ CBL is reconstructed only by diffusion, while for *Ra* ≥ (*Ra*)_c_ diffusion reconstructs CBL and simultaneously hydrodynamic instabilities cause a blur of CBL by gravitational convective stirring of solutions. The model based on Eqs. (5)–(8) and (11) allows to calculate the concentration Rayleigh number of CBL (*Ra*) during diffusive CBLs buildup. Gravitational convective stirring of solution in CBL appears at the moment in which *Ra* reaches its critical value (*t* = *T*_o_ for *Ra* = (*Ra*)_c_). *T*_o_ is the characteristic parameter of conditions of appearance of gravitational convection in the membrane system [21].

## Experiment

The measurements were carried out in the system with bacterial cellulose membrane (*Biofill*), oriented in a horizontal plane, between the two chambers with a volume of 1.75 × 10^−4^ m^3^ each, filled with aqueous solutions of KCl. The membrane system was presented [17]. Transport properties of bacterial cellulose membrane (*Biofill*) for KCl are determined by the following coefficients: diffusion permeability coefficient *ω*_*s*_ = 1.94 × 10^–9^ mol N^−1^ s^−1^, hydraulic permeability coefficient *L*_*p*_ = 6.5 × 10^−11^ m^3^ N^−1^ s^−1^, and reflection coefficient *σ* = 0.0034. Values of these coefficients were measured experimentally by methods described in [26].

**Fig. 1:**
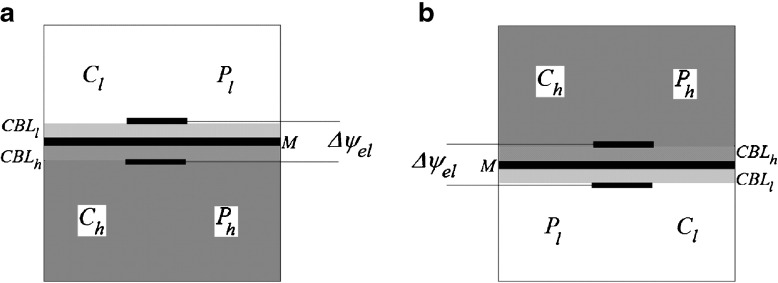
The membrane system: *M* membrane, *CBL* concentration boundary layer, *C*_*h*_, *C*_*l*_ – KCl concentrations, *P*_*h*_, *P*_*l*_ – mechanical pressures in chambers, (*h* – higher, *l* - lower), Δ*ψ*_*el*_ – difference of potentials between Ag|AgCl electrodes. Configurations of the membrane system A (a) and B (b)

Figure 1 shows possible configurations of the membrane system during measurement of voltage in the membrane system. Configuration A (Fig. 1a) of the membrane system contains KCl solutions with higher density (higher concentration - *C*_*h*_) under the membrane. In turn, configuration B (Fig. 1b) contains KCl solutions with lower density (lower concentration - *C*_*l*_) under the membrane. KCl solutions have greater density for solutions with greater KCl concentration.

In configuration A of the membrane system only diffusion is the cause of CBL buildup because the density gradient appearing in CBLs is directed as gravitational acceleration. In turn, in B configuration of the membrane system diffusion reconstructs CBLs in such a way that gradient of solution density appearing in CBLs is directed oppositely to gravitational acceleration. For sufficiently high value of density gradient in CBL, the movement of solution with higher density downward and solution with lower density upward causes gravitational convective stirring of solutions in near membrane areas. This results in a blur of CBLs and the configuration B is hydrodynamically unstable.

During CBLs buildup, the voltage between electrodes immersed in solutions on both sides of the membrane decreases in time. In configuration B of the membrane system, with sufficiently high difference of KCl concentrations in chambers at the initial moment, we observed the appearance of pulsations of measured voltage [1]. The dynamics of CBL buildup, and its transition from diffusive to diffusive-convective conditions, is characterized by the time preceding the onset of voltage oscillations (*T*_o_). At the initial moment in each experiment, the lower KCl concentration amounted to *C*_*l*_ *=* 0.01 mol m^−3^, while the higher concentration *C*_*h*_ amounted to 1 mol m^−3^ or 10 mol m^−3^. The area of the membrane under investigation was 3.1 × 10^−4^ m^2^. In order to assure homogeneity of solutions at the initial moment, the mechanical stirring has been used at a rate of 500 rpm. Measurement of voltage between the electrodes in the membrane system is initiated when the stirring of the solutions is terminated. The difference of pressure (Δ*P*) fixed on the membrane during measurement of voltage between electrodes in the membrane system was in the range from Δ*P* = –40 kPa to Δ*P* = +40 kPa and was stabilized during measurements [17]. A pump generated mechanical pressure difference on the membrane, controlled and stabilized by a manometer. The error of pressure difference stabilization was lower than 5%. We accepted a negative value of Δ*P* for the case with higher pressure in the chamber with lower KCl concentration. In this case, Δ*P* on the membrane was oppositely directed to the osmotic pressure difference (Δπ).

Voltage between electrodes was measured by means of Ag|AgCl electrodes connected with voltmeter (MERATRONIK Type U726) and a computer. The electrodes were located on both sides of the membrane and the distance of each electrode from the membrane equaled 6 mm. The input impedance of the voltmeter was to 0.1 GΩ, and its accuracy was 0.1 mV. The membrane system was thermostated and enclosed by a Faraday cage in order to minimize electrical interference. The temperature of solutions was 295 ± 0.5 K. The chambers of the membrane system were filled with solutions, stirred by magnetic stirrers until the voltage was settled, no longer than 1–2 min. After turning off the mechanical stirring, the voltage was measured every 2 s for 300 min. The error of preparation of solution concentrations was lower than 1.5%, while relative error of voltage measurements for those same initial conditions was lower than 5%.

## Results and discussion

We used Eqs. (5), (10), and (11) to model KCl transport through the bacterial cellulose membrane and CBLs. For this purpose, we used MathCad Prime 3.0 with layer thickness *d*_*w*_ = 10^–4^ m, time interval Δ*t* = 1 s, the diffusion coefficient of KCl in aqueous solution *D*_*s*_ *=* 2.01 × 10^−9^ m^2^ s^−1^, *T* = 295 K, *R* = 8.31 J mol^–1^ K^–1^, water kinematic viscosity ν = 0.892 ⋅ 10^−6^ m^2^ s^−1^, change of KCl solution density with concentration $\frac{\partial \rho}{\partial C}=0.0436\kern0.6em \mathrm{kg}\;\mathrm{m}\mathrm{o}{\mathrm{l}}^{-1}$, coefficients for bacterial cellulose membrane: diffusion permeability coefficient *ω*_*s*_ = 1.94 × 10^–9^ mol N^−1^s^−1^, the hydraulic permeability coefficient *L*_*p*_ = 6.5 × 10^–11^ m^3^ N^−1^ s^−1^ and reflection coefficient *σ* = 0.0034. Besides, we assumed in the model that KCl solutions in chambers initially are homogeneous and that the membrane surface is equal to the cross-sectional area of chambers. At initial moment the concentrations in layers fulfil conditions *C*_2,*n*_^0^ = *C*_*l*_ and *C*_1,*n*_^0^ = *C*_*h*_ for all n. The initial lower concentration (*C*_*l*_) was corrected according to the procedure described in the article [17].

### Configuration A of the membrane system

**Fig. 2:**
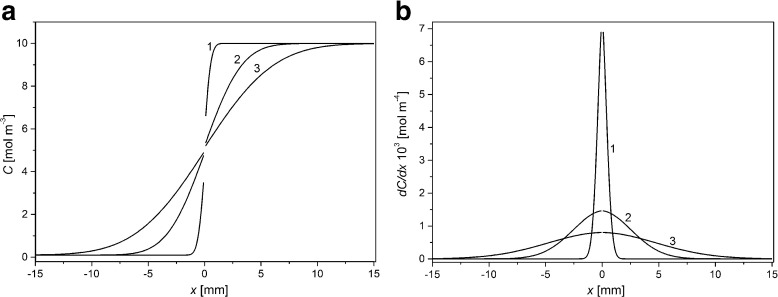
Distribution of KCl concentration (**a**) and gradient of KCl concentration (**b**) in chambers of the membrane system, for ΔP = 0 kPa and for times of CBLs buildup: 1 min (1), 30 min (2), and 100 min (3). *C*_h_/*C*_l_ = 1000

Based on Eqs. (5)–(7), we counted the distribution of concentrations and gradient of concentrations near membrane surfaces during CBLs buildup. Figure 2 shows KCl concentration (a) and gradient of KCl concentration (b) as functions of distance from the membrane surfaces (*x*) for ΔP = 0 kPa and for times of CBLs buildup: 1 min (1), 30 min (2), and 100 min (3) respectively. We assumed that for the chamber with lower KCl concentration (solution over the membrane) the distance from the membrane is negative, while for the chamber with greater KCl concentration it is positive. The location of the membrane is for *x* = 0. The initial *C*_h_/*C*_l_ was equal to 1000.

For ΔP = 0 kPa CBLs, thicknesses in both chambers are the same and maximal gradients of KCl concentration are observed at membrane surfaces. An increase of time of CBL buildup causes an increase of CBL thicknesses and a decrease of amplitude of peak, while the location of the peak during CBLs buildup is on the membrane.

**Fig. 3:**
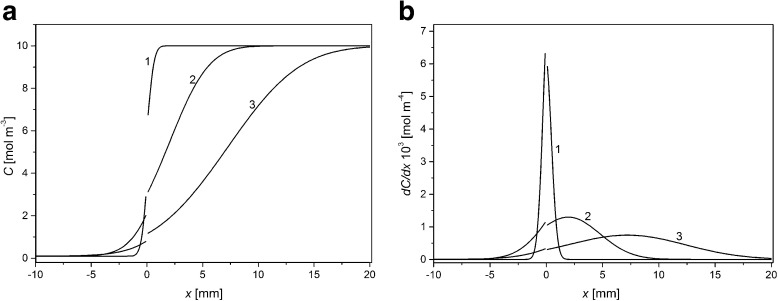
Distribution of KCl concentration (**a**) and gradient of KCl concentration (**b**) in chambers of the membrane system for ΔP = –20 kPa and for times of CBLs buildup: 1 min (1), 30 min (2), and 100 min (3). *C*_h_/*C*_l_ =1000

Figure 3 shows KCl concentration (a) and gradient of KCl concentration (b) as functions of distance from the membrane surfaces for ΔP = − 20 kPa and for times of CBLs buildup: 1 min (1), 30 min (2), and 100 min (3) respectively.

For ΔP lower than zero concentration and gradient of concentration distribution are asymmetrical in relation to the membrane (x = 0). CBL thickness in the chamber with higher concentration is greater than CBL thickness in the chamber with lower concentration. This may be called “compression” of CBL in the chamber with lower concentration and simultaneous “enlargement” of CBL in the chamber with a higher concentration (change of CBL thickness in comparison with the case of ΔP = 0 kPa). For ΔP < 0 and longer times of CBLs buildup, the maximum of gradient of concentration is lower and shifted further into the chamber with higher KCl concentration.

**Fig. 4:**
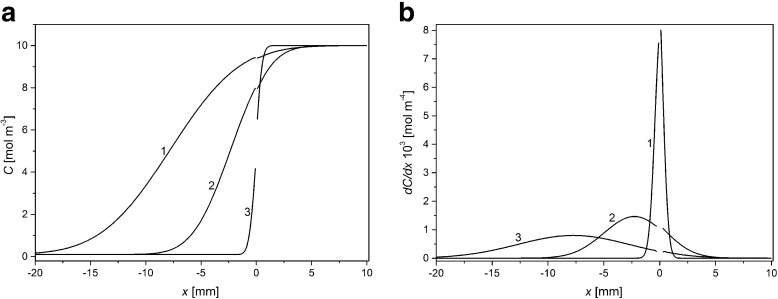
Distribution of KCL concentration (**a**) and gradient of KCl concentration (**b**) in chambers of the membrane system for ΔP = +20 kPa and for times of CBLs buildup: 1 min (1), 30 min (2), and 100 min (3). *C*_h_/*C*_l_ =1000

Figure 4 shows KCl concentration (a) and gradient of KCl concentration (b) as functions of distance from the membrane surfaces for ΔP = +20 kPa and for times of CBLs buildup: 1 min (1), 30 min (2), and 100 min (3).

For ΔP > 0, greater flux of solute through the membrane compared with the case ΔP = 0 causes asymmetry of CBL thicknesses. CBL in the upper chamber with lower concentration is greater than CBL thickness in the lower chamber with higher concentration. The maximum of gradient of KCl concentration in CBL decreases with time and is shifted farther from the membrane into the chamber with lower KCl concentration. Peak amplitudes for ΔP = 0 kPa and ΔP = +20 kPa are similar while for ΔP = –20 kPa amplitude of peak is smaller by about 20% than for ΔP = 0 kPa.

**Fig. 5:**
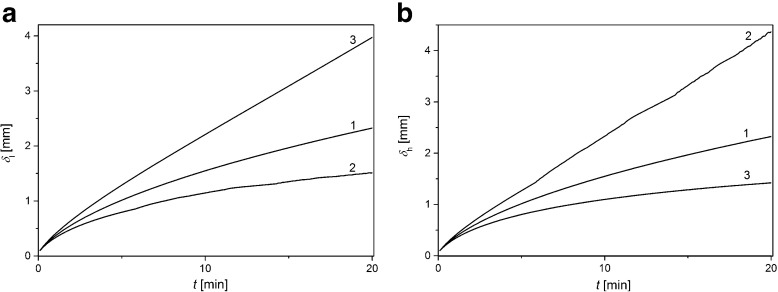
CBL thicknesses as functions of time of CBLs buildup in chamber with lower (**a**) and higher (**b**) concentrations for ΔP equal to: 0 kPa (1) +20 kPa (2), and −20 kPa (3). *C*_h_/*C*_l_ =1000

Figure 5 shows CBL thickness in the chamber with lower (a) and higher (b) KCl concentrations as functions of time of CBLs buildup, calculated from Eqs. (5)–(8)

For ΔP = 0 kPa, CBL thickness grows nonlinearly in time and is equal in both chambers (graphs 1 in Fig. 5a and b). For ΔP > 0, increase of CBL thickness in the chamber with lower concentration (graph 2 Fig. 5a) is slower in time, while in chamber with higher concentration (graph 2 Fig. 5b) is faster than in the case of ΔP = 0 kPa. The changes in time of CBL thickness for ΔP < 0 kPa (graphs 3 in Figs. 5a and 5b) are inverted in comparison with ΔP > 0 kPa. This indicates that the application of pressure difference through the membrane entails the asymmetry of CBLs buildup.

**Fig. 6:**
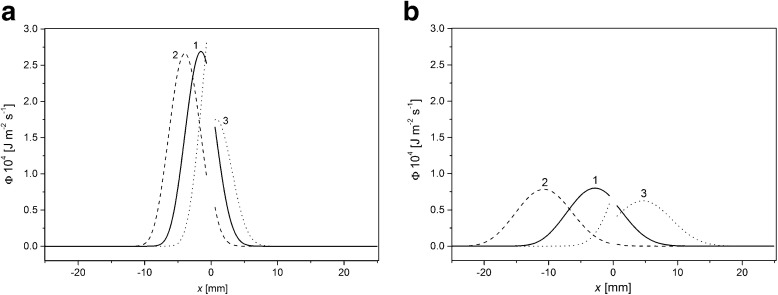
The dissipation energy in CBLs as a function of distance from the membrane surface for times of CBLs buildup: 30 min (**a**) and 100 min (**b**) and for ΔP equal to: 0 kPa (1 - *continuous line*), +20 kPa (2 - *dashed line*) and −20 kPa (3 - *dotted line*). *C*_h_/*C*_l_ =1000

The next important parameter is dissipation energy during transport of solutes through the chambers. We calculated dissipation energy based on Eqs. (5)–(7) and (10). Figure 6 shows the results of calculations of energy dissipated in one second as a function of distance from the membrane surface, for ΔP equal to: 0 kPa (1), +20 kPa (2) and −20 kPa (3) and for time of CBLs buildup: 30 min (a) and 100 min (b) respectively.

The main feature of dependence of dissipation energy on distance from the membrane is its maximal value. Location and amplitude of this peak depend on time of CBLs buildup and ΔP. In contrast to the case of dependence of gradient of concentration as a function of distance from the membrane (Fig. 2b) the peak of dependence Φ = *f*(*x*) for ΔP = 0 kPa is located in chamber with lower concentration (*x* = – 2 mm, graph 1 in Fig. 6a). For ΔP > 0, the distance of the peak from the membrane is greater than for ΔP = 0, while for ΔP < 0 kPa is lower and for ΔP = –20 kPa the maximum of dissipation energy is observed in the chamber with higher concentration. After 30 min of CBLs buildup and for ΔP > 0, the amplitude of the peak is slightly lower than in the case of ΔP = 0 kPa, while for ΔP < 0 the amplitude of the peak is smaller by about 35%. Increase of time of CBLs buildup for all ΔP causes decrease of peak amplitude and shift of this maximum further into the chamber with lower pressure.

We also calculated dissipation energy in In both chambers: ${\varPhi}_l=\frac{1}{l_c}{\displaystyle \underset{-{l}_c}{\overset{o}{\int }}\varPhi (x) dx}$, ${\varPhi}_h=\frac{1}{l_c}{\displaystyle \underset{0}{\overset{l_c}{\int }}\varPhi (x) dx}$, where *l*_*c*_ is the length of the chamber. We also calculated dissipation energy in membrane (*Φ*_*m*_) based on equation

12 $$ {\varPhi}_m={J}_v\cdot \varDelta p+{J}_{sd}\varDelta {\pi}_m $$

where *J*_*v*_ is the volume flux through the membrane, ${J}_{s d}=\frac{1}{\overline{C}}{J}_s-{J}_v{V}_w$ is the diffusive flux through the membrane , $\overline{C}$ is the average solute concentration in the membrane and *V*_*w*_ is the partial volume of water. *Φ*_*tot*_ = *Φ*_*l*_ + *Φ*_*h*_ + *Φ*_*m*_. Table 1 presents the results of the calculations.

**Table 1:** Total dissipation energy in each chamber (Φ_l_ , Φ_h_) and dissipation energy in membrane (Φ_m_) calculated for times of CBLs buildup: 30 min and 100 min and for ΔP equal to: 0 kPa, +20 kPa, and −20 kPa

	t = 30 min				t = 100 min			
Δp	*Φ*_*h*_	*Φ*_*l*_	*Φ*_*m*_	*Φ*_*tot*_	*Φ*_*h*_	*Φ*_*l*_	*Φ*_*m*_	*Φ*_*tot*_
[kPa]	[mW m^−2^]				[mW m^−2^]			
−20	5.79 (14%)	5.37 (13%)	30.15 (73%)	41.31	5.67 (17%)	1.15 (3%)	27.05 (80%)	33.87
0	2.90 (19%)	10.53 (69%)	1.92 (12%)	15.35	1.79 (2.5%)	5.94 (71.5%)	0.579 (7%)	8.309
20	0.627 (1.5%)	14.33 (34.5%)	26.56 (64%)	41.52	0,037 (0.1%)	8,17 (23,9%)	26.08 (76%)	34.28

As results from Table 1, in the case of ΔP = 0 kPa the major part of total energy *Φ*_*tot*_ (about 70%) is dissipated in the chamber with lower concentration. The data presented in Table 1 show that in the absence of a pressure difference across the membrane, a substantial part of the energy (nearly 90%) is dissipated in the chambers. An increase in the difference of mechanical pressure through the membrane causes the amount of energy dissipated in the membrane to increase and for ΔP = +20 kPa (or −20 kPa) more than 70% of energy is dissipated in the membrane. An increase of time of CBLs buildup causes a decrease of all considered dissipation energies.

### Configuration B of the membrane system

**Fig. 7:**
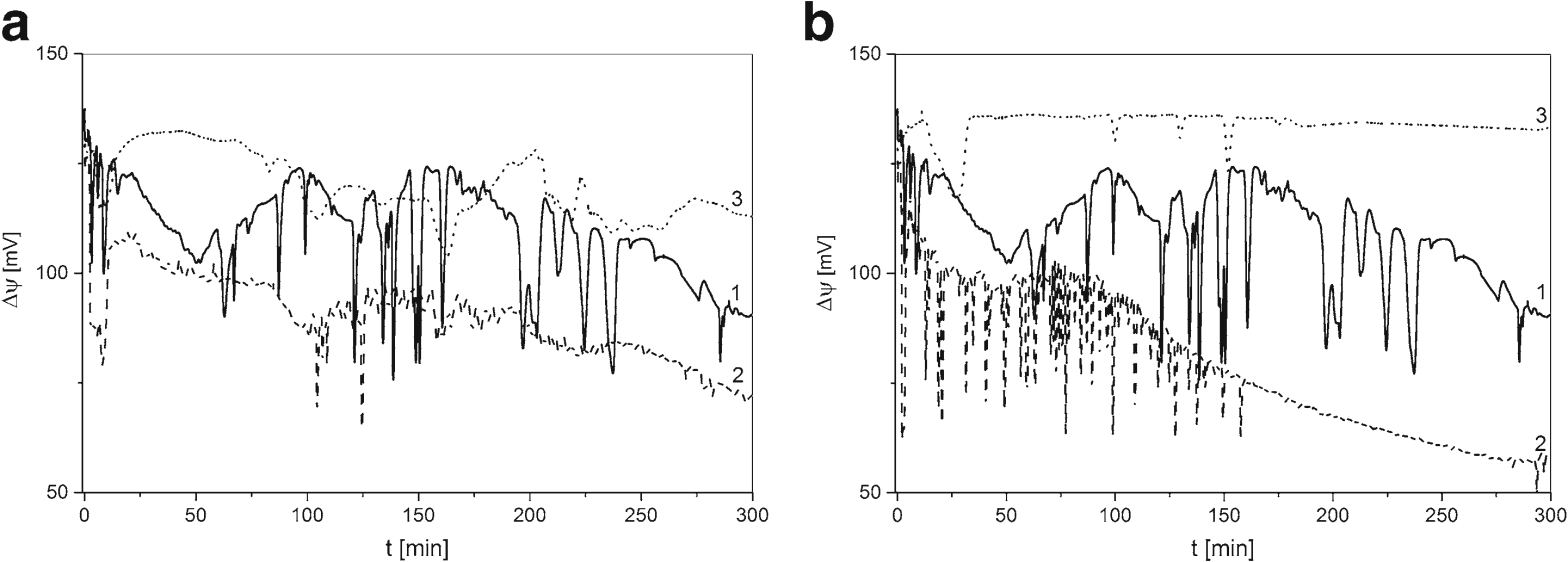
Voltage measured in the membrane system in configuration B as a function of time for ΔP equal to: 0 kPa (a-1,b-1), +20 kPa (a-2), −20 kPa (a-3), +40 kPa (b-2), and −40 kPa (b-3). Initial quotient of concentrations in chambers *C*_h_/*C*_l_ = 1000

Figure 7 shows time dependencies of voltage measured between Ag|AgCl electrodes in configuration B of the membrane system for ΔP equal to: 0 kPa (1), +20 kPa (1), and −20 kPa (3) (Fig. 7a) and 0 kPa (1), +40 kPa (2), and −40 kPa (3) (Fig. 7b).

Analyzing Fig. 7a and b, we can identify pulsations of voltages caused by gravitational stirring of solutions in chambers. In B configuration of the membrane system, density gradients appearing in CBLs are directed oppositely to gravitational acceleration. For sufficiently high density gradients, hydrodynamic instabilities appear in near-membrane areas causing gravitational stirring of solutions in chambers. Amplitudes and frequencies of pulsations depend on mechanical pressure difference across the membrane. When this difference is greater than zero, time characteristics of measured voltages (Fig. 7, a-2 and b-2) are shifted into lower values of voltage in comparison to ΔP = 0 kPa (Fig. 7, a-1 and b-1). For ΔP = +40 kPa, the amplitude of pulsations falls to nearly zero after 150 min of CBLs buildup. On the contrary, a mechanical pressure difference lower than zero causes a shift of time characteristics of measured voltages (Fig. 7, a-3 and b-3) into higher values with substantial decrease of frequency and amplitude of pulsations. The pulsations of measured voltages are mainly related to concentration changes near the electrode in the chamber with lower concentration because of greater relative changes of KCl concentration near this electrode in comparison with relative changes of KCl concentration near the electrode in the chamber with higher concentration. When ΔP is directed as the difference of osmotic pressure (ΔP > 0), a faster increase of CBL in the chamber with lower concentration causes greater and faster changes of measured voltages. On the contrary, for ΔP < 0 “compression” of CBL in the chamber with lower concentration causes smaller changes of measured voltages in time of CBLs buildup.

**Fig. 8:**
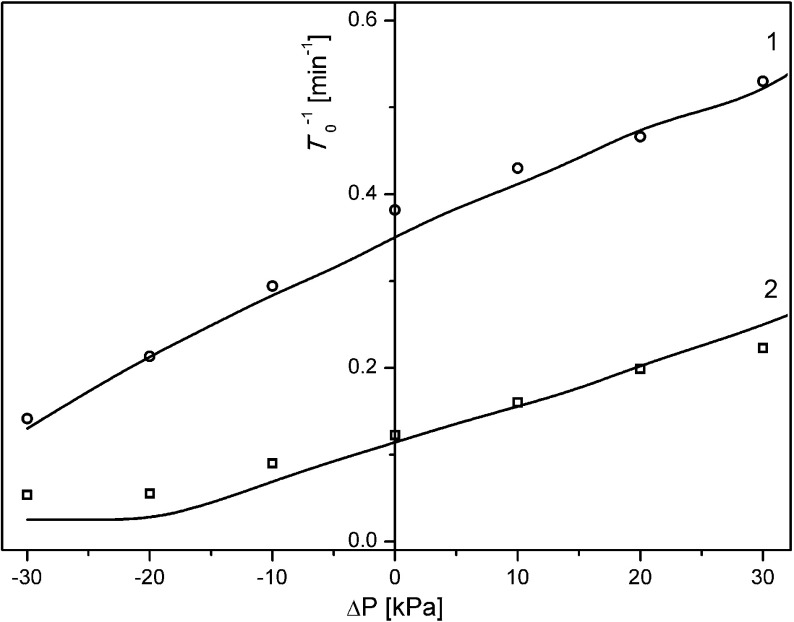
Inverse of time of appearance of pulsations of voltage (*T*_o_^−1^) as a function of mechanical pressure difference on the membrane for data from experiment (*points*) and from the model (*lines*) respectively for *C*_h_/*C*_l_ = 1000 (1) and *C*_h_/*C*_l_ = 100 (2)

Analyzing the first few minutes of time characteristics of voltage between electrodes in the membrane system we can define the initial moment of appearance of pulsations (marked by *T*_o_) [21]. For Ra < (*Ra*)_*c*_, we observe diffusional CBLs buildup (smooth decrease of voltage) while for Ra > (*Ra*)_*c*_ we observe hydrodynamic instabilities visible as pulsations of measured voltage. Based on Eqs. (5), criterion (8) and Rayleigh concentration number (11), we calculated time of appearance of gravitational convection in the chamber with lower KCl concentration. We used the critical value of Rayleigh number (*Ra*)_*c*_ as the condition for appearance of hydrodynamic instabilities. This approach gives good agreement with experimental data and is caused, as was previously written, by greater relative changes of KCl concentration near the electrode in the chamber with lower concentration during CBLs buildup than near the electrode in the chamber with higher concentration. The moment of appearance of gravitational convection calculated for CBL in the chamber with higher concentration does not agree with the experiment, so the voltage pulsations (moment of appearance, amplitude, and frequency) are mainly connected with concentration changes near the electrode in the chamber with lower concentration. Figure 8 shows the inverse of time of appearance of voltage pulsations (*T*_o_) as a function of difference of mechanical pressure on the membrane for data from experiment (points) and from model (line) respectively for initial quotients of concentrations in chambers: *C*_h_/*C*_l_ = 1000 (1) and *C*_h_/*C*_l_ = 100 (2).

As results from Fig. 8 the dependence *T*_o_^−1^ = *f*(ΔP) is nearly linear in studied range of ΔP and for ΔP < 0 *T*_o_^−1^ is lower than for ΔP > 0. An increase of initial quotient of concentrations in the membrane system at initial moment causes a decrease of *T*_o_ for all analyzed ΔP (shift of *T*_o_^−1^ = *f*(ΔP) into higher values in whole range of ΔP). For ΔP greater than zero, CBL in the chamber with lower concentration is reconstructed faster than for ΔP = 0 kPa. This results in appearance in shorter time a sufficiently large density gradient in CBL, to initiate convective stirring of solution in CBL, observed as pulsations of measured voltage. For ΔP < 0, CBL in the chamber with lower concentration is reconstructed in time slower than for ΔP = 0 kPa, so the time needed for appearance of gravitational convective stirring of solution in this CBL (observed as pulsations of voltage) is longer (lower values of *T*_o_^−1^).

## Conclusions

1. The concentration and gradient of concentration in CBLs as functions of distance from the membrane for zero pressure difference on the membrane is symmetrical. The maximum of gradient of concentration in CBLs is observed at membrane surfaces. An increase of time of CBLs buildup causes a decrease of maximal value of this gradient.
2. For ΔP > 0, maximum of concentration gradient and greater CBL thickness are observed in the chamber with lower concentration while for ΔP < 0 in the chamber with higher concentration. An increase of time of CBLs buildup causes a nonlinear decrease of maximum of concentration gradient and a nonlinear increase of CBL thicknesses.
3. The maximum of dissipation energy in CBLs depends on the mechanical pressure difference on the membrane and time of CBLs buildup. For ΔP = 0, this maximum is located in the chamber with lower KCl concentration.
4. The application of mechanical pressure difference on the membrane in configuration B of the membrane system substantially changes time characteristics of voltages measured in the membrane system, changing frequency, amplitude of voltage pulsations, and the moment of appearance of pulsations caused by gravitational convective stirring of the solution. Pulsations of voltage are associated with sufficiently high gradients of density in CBLs, oppositely directed to gravitational acceleration.
5. The time needed for appearance of gravitational convective stirring of solutions in CBL (*T*_o_) for this same initial osmotic pressure difference on the membrane at initial moment depends on the mechanical pressure difference on the membrane. *T*_o_^−1^ is almost a linearly increasing function of ΔP in the studied range of mechanical pressure difference on the membrane. An increase of initial osmotic pressure difference on the membrane causes a decrease of *T*_o_ in the whole range of studied ΔP.

## Nomenclature

*J*_*v*_: Volume flux through the membrane
*J*_*s*_: Solute flux through the membrane
*ΔP* = *P*_*h*_ − *P*_*l*_: Mechanical pressure difference across the membrane
*Δπ*_*s*_ = *RT*(*C*_*h*_ − *C*_*l*_): Osmotic pressure difference across the membrane
${\overline{C}}_s=\left({C}_h-{C}_l\right){\left[ \ln \left({C}_h{C_l}^{-1}\right)\right]}^{-1}$: An average solute concentration in the membrane
*C*_*h*_,*C*_*l*_: Solute concentrations in chambers under (*h* - higher concentration) and over (*l* - lower concentration) the membrane
*C*_*l*,*n*_^*k*^,*C*_*h*,*n*_^*k*^: Concentrations in layers (*n*) near membrane in time *t = k*, in chambers with lower (*l*) or higher (*h*) solute concentration
*L*_*p*_: Hydraulic permeability coefficient of the membrane [m^3^ N^−1^ s^−1^]
*σ*_*s*_: Solute reflection coefficient
*ω*_*s*_: Solute permeability coefficient [mol N^−1^ s^−1^]
*κ*: Conductivity of the membrane
*D*_*s*_: Diffusion coefficient of solute in aqueous solution
*t*_*s*_: Transference number of solute *s*
*F*: Faraday number
*R*: Gas constant
*Ra*: Concentration Rayleigh number
*T*: Temperature [K]
Φ: Dissipation energy
